# Development and evaluation of a 3-D virtual pronunciation tutor for children with autism spectrum disorders

**DOI:** 10.1371/journal.pone.0210858

**Published:** 2019-01-28

**Authors:** Fei Chen, Lan Wang, Gang Peng, Nan Yan, Xiaojie Pan

**Affiliations:** 1 CAS Key Laboratory of Human-Machine Intelligence-Synergy Systems, Shenzhen Institutes of Advanced Technology, Shenzhen, China; 2 Department of Chinese and Bilingual Studies, The Hong Kong Polytechnic University, Hong Kong Special Administrative Region; 3 Shenzhen Aixin Zhihui Rehabilitation Centre for Children with Special Needs, Shenzhen, China; Vita-Salute San Raffaele University, ITALY

## Abstract

The deficit in speech sound production in some children with autism spectrum disorder (ASD) adds to their communication barriers. The 3-D virtual environments have been implemented to improve their communication abilities. However, there were no previous studies on the use of a 3-D virtual pronunciation tutor designed specifically to train pronunciation for children with ASD. To fill this research gap, the current study developed and evaluated a 3-D virtual tutor which served as a multimodal and real-data-driven speech production tutor to present both places and manners of Mandarin articulation. Using an eye-tracking technique (RED 5 Eye Tracker), Experiment 1 objectively investigated children’s gauged attention distribution online while learning with our computer-assisted 3-D virtual tutor in comparison to a real human face (HF) tutor. Eye-tracking results indicated most participants showed more interests in the visual speech cues of the 3-D tutor, and paid some degree of absolute attention to the additional visual speech information of both articulatory movements and airflow changes. To further compare treatment outcomes, training performance was evaluated in Experiment 2 with the ASD learners divided into two groups, with one group learning from the HF tutor and the other from the 3-D tutor (HF group vs. 3-D group). Both groups showed improvement with the help of computer-based training in the post-intervention test based on the calculation of a 5-point Likert scale. However, the 3-D group showed much higher gains in producing Mandarin stop and affricate consonants, and apical vowels. We conclude that our 3-D virtual imitation intervention system provides an effective approach of audiovisual pronunciation training for children with ASD.

## Introduction

Given the high incidence of autism spectrum disorder (ASD), at close to 1% worldwide [1], there is an urgent need to improve our understanding of ASD and to refine our treatment strategies. Besides the social communication deficits and repetitive sensory-motor behaviors, speech and language disorders tend to be a hallmark of ASD. Among the communicative characteristics of children in the second and third year of life who are identified with ASD, delayed onset and development of the spoken language tends to be one of the key signs and symptoms. Approximately 10–25% of all children with ASD fail to develop speech to communicate with others [2]. Due to the known deficits that characterize ASD in the theory of mind [3], researchers have identified that pragmatic skill is the most seriously impaired in terms of language deficits in ASD [4,5]. In contrast, less attention has been paid to the articulatory and phonological deficits among this population.

Articulation skills were sometimes reported to be relatively intact, or even better, when compared to other speech and language behaviors in individuals with ASD [6–8]. However, in these studies, articulation tests were only judged as correct or incorrect, giving no information on the nature of the pronunciation errors. Other studies, using more detailed phonological analyses, have detected more severe speech sound impairment in individuals with ASD [9–11]. Recently, one study has reported that approximately 41% of school-aged participants with ASD in their research produced some pronunciation errors [12]. To conclude, although there remains some disagreement in the literature on the status of phonological development in an ASD cohort, relevant research overall indicates that at least a subgroup of children with ASD may exhibit speech sound production difficulties. Moreover, several studies [13–16] have found a strong relationship between the severity of pronunciation difficulty and the severity of overall language impairment, suggesting that some children with ASD who present with more severe global language impairment may also exhibit more severe speech production difficulties.

Appropriate communication depends on the use of a vast array of language skills (e.g., phonology, lexicon, grammar, semantics, and pragmatics), with phonological decoding and encoding acting as the initial and final operations of the input-processing-output language system. Articulatory and phonological difficulties can affect speech intelligibility to some extent, and they represent an additional social and communication barrier for people with ASD. One study [12] highlighted the contribution of speech sound errors to communication barriers in ASD, even when only a few pronunciation errors occurred. Furthermore, some studies [11,12] have emphasized that there was no correlation between chronological age and number of speech errors, suggesting that speech distortions did not appear to resolve themselves over time in individuals with ASD. Thus, unlike typically developing (TD) children who refine their speech production skill through accumulated language experience with age [17], pronunciation difficulties for some children with ASD may continue into adulthood if there is no effective intervention. Consequently, speech production difficulties should be treated early in children with ASD, and the inclusion of the phonological component in treatment should be applied [15,18].

However, there have been few empirical reports so far on treatment strategies specifically targeted at enhancing speech production skill among the ASD population. There is an overall dearth of knowledge concerning articulatory interventions among children with ASD. A review article [19] found a very limited number of articles concerning ASD and phonological interventions. For example, one study [20] emphasized stimulating learning motivation in children with ASD. Later, other studies on the treatment of speech sound production focused mainly on improving teaching strategies of therapist [2,21,22]. These studies on phonological interventions were conducted among a relatively small sample size, with single case studies [21,22] or in three ASD children [2]. Since there were no controls for individual differences and other variables, it is difficult to make generalizations from these studies. Recently, a novel intonation-based intervention called Auditory-Motor Mapping Training has been developed to facilitate speech output in non-verbal children with ASD [23]. After therapy, all the six non-verbal children showed significant improvements in their ability to articulate several word approximations. Although this training approach showed great success in promoting the production of syllable approximations in ASD children who are non-verbal, it did not focus on improving fine-grained production of speech sound elements.

Recently, several novel approaches have been proposed to help ameliorate communication deficits in children with ASD. Previous studies on the role of the mirror neuron system (MNS) and the observed links between the MNS and imitation suggested that mirror system based imitation learning could be used as an efficacious treatment strategy for children with ASD [24,25]. Moreover, another highly promising technique was to present the 3-D virtual environments to the teaching-learning process for children with ASD [26]. Virtual reality (VR), i.e. a simulation of the real world based on computer graphics, has recently emerged in many domains of rehabilitation in children with ASD, and this technique can be useful since it allows teachers or therapists to present a repeatable and diversifiable environment during learning in a very similar context to the real ones in the absence of potential risks [27]. With the progress of speech technology, various 3-D virtual tutors have been utilized as new ways to act as one of the non-immersive desktop VRs and directly show learners with pronunciation animations for imitation learning. Several reasons explain why the use of auditory and visual information from a 3-D tutor is so successful, and why it holds so much promise for pronunciation tutoring [28]. Both internal and external articulatory movements have been demonstrated in 3-D virtual tutors to successfully guide the pronunciation training for hearing-loss children [29–31] and second language learners [32,33]. Recently, a computer-animated talking head has been employed to train and develop vocabulary and grammar knowledge for children with ASD [34,35]. However, to the best of our knowledge, until now, there have been no studies on the use of a 3-D virtual pronunciation tutor designed specifically to enhance speech production skills among the ASD population.

The underlying sources of speech delays/disorders in children with ASD have important implications for the rationale of using a 3-D virtual pronunciation tutor for speech therapy in ASD. Many reasons may account for the speech sound production difficulties experienced by children with ASD. As indicated by the ‘social theory’ [3], some studies pointed out that failure to attend to the ambient social language environment during daily communication negatively impacts the ability to acquire spoken language in children with ASD [11,36,37]. Probably, the utilization of a 3-D virtual tutor presented on a computer screen has the potential to reduce the social learning burden for ASD learners by avoiding communication with a real human teacher. Furthermore, it is important to note that our 3-D virtual tutor, compared to real human face tutor, contains additional visual information with a profile view (such as internal articulator and airflow animations). Given that speech perception and production are presumed to be closely correlated constructs, impaired or atypical speech sound perception in the auditory modality [38,39] may be another factor leading to speech production deficits in children with ASD. Also, the traditional pronunciation training approach mainly with the help of auditory inputs may not be efficacious enough to enhance speech production in ASD learners. Hopefully, the additional visual speech cues in our 3-D virtual tutor, if noticed by ASD learners, may offer supplementary production guidance for intended imitation learning from the visual modality.

The overall goal of this study is to utilize recent advances in speech technology to develop and evaluate a 3-D virtual tutor for pronunciation training in children with ASD. We first introduced some of our previous studies on the development of a 3-D virtual tutor showing Mandarin articulatory and aspiratory animations [29,40–42]. This speech production tutor has been developed to produce articulatory movements in accordance with airflow motions when uttering Mandarin syllables. We then evaluated this 3-D virtual pronunciation tutor with an eye-tracking study (Experiment 1) and a pronunciation training study (Experiment 2), to ascertain whether the 3-D virtual tutor is effective in improving the level of the ASD learners’ interest and enhancing the accuracy of their pronunciation of Mandarin consonants and vowels.

For Experiment 1, the objective evaluation of our 3-D pronunciation tutor is the first focus of this study since a virtual tutor system involved in any application must first undergo evaluation. The uncanny valley effect [43] suggested that the learners' familiarity with language training systems with the same underlying speech model could differ significantly. The transparent face and the cooperative motions of various internal articulators and airflow in our 3-D tutor are not commonly seen in the children’s daily lives. It is unclear whether children with ASD would pay attention to these uncommon visual contents. The eye-tracking approach allows for objective and quantitative observation of attention, and through the analysis of fixation patterns, can indicate which information from a tutor is available to the learner [44]. Because it is non-invasive and does not require advanced motor responses or language, eye tracking is particularly suitable for conducting studies in young children with ASD. In Experiment 1, by using an eye-tracking methodology, our 3-D virtual pronunciation tutor was evaluated in comparison to real human face videos. The pronunciation tutors were shown through a computer screen under two conditions: real human face tutor (HF tutor) and 3-D pronunciation tutor (3-D tutor), each with a front view first and a profile view afterwards. By analyzing eye-tracking related measures, three research questions (RQs) were put forward and investigated:

RQ1: When presented with visual speech cues, which tutor is more attractive to children with ASD, HF tutor or 3-D tutor?
RQ2: For the 3-D tutor with a profile view, did children with ASD pay attention to the uncommon visual contents containing internal articulator and airflow motions?
RQ3: Compared with age-matched TD children, did children with ASD show a similar attention pattern while watching the pronunciation tutors?

It is hypothesized that, due to their attention deficits [45], children with ASD might show a much more scattered attention pattern compared with TD children. Moreover, since children with ASD tended to show a processing deficit towards real human faces [46], they might exhibit more interests in the visual speech cues of 3-D virtual tutor, and pay some degree of visual attention to the uncommon visual contents (internal articulator and airflow animations) in our 3-D tutor with a profile view.

For Experiment 2, in order to further evaluate the efficacy of our 3-D virtual tutor as a pronunciation training tool for the acquisition of Mandarin speech, a pronunciation training study was thus conducted to compare pronunciation performance between two subgroups of children with ASD, learning from HF tutors or 3-D pronunciation tutors respectively (HF group vs. 3-D group). As our 3-D virtual tutor contains real-data-driven visual speech cues which offer realistic pronunciation guidance from the visual modality, it is hypothesized that the 3-D tutor might be more effective in facilitating the imitation learning of Mandarin consonants and vowels in ASD learners.

## Materials and methods

### Development of a 3-D virtual pronunciation tutor

The visual speech cues in a 3-D virtual tutor may offer supplementary production guidance from the visual modality. Our 3-D virtual tutor was presented as an entire-head virtual tutor on a computer screen, showing models of the face, lips, tongue, jaw, and nasopharyngeal wall (based on MRI data). Moreover, the internal articulator (i.e., tongue) and airflow changes in our 3-D tutor with a profile view were animated based on physiological signals during pronunciation, in order to generate a more realistic pronunciation tutor. In this way, the ‘audio-visual’ MNS [47] may be activated to facilitate intended imitation learning from the internal articulatory model and airflow model. Furthermore, since our 3-D virtual pronunciation tutor targeted especially at enhancing speech sounds (vowels and consonants) production in Mandarin-speaking children with ASD, the phonology system in Mandarin should also be considered. Both the internal articulatory model and airflow model were concurrently implemented in our 3-D virtual tutor with a profile view to offer realistic visual speech cues (i.e., viseme, the visual equivalent of a phoneme or unit of sound in spoken language) to ASD learners. During the training program, the additional visual information in our 3-D virtual imitation intervention system was exhibited to ASD learners for imitation learning of pronunciation.

First, to realize the articulatory model, the external and internal articulatory movements of each syllable were collected and recorded by an Electro-Magnetic Articulography (EMA AG500). The 3-D articulation data were recorded from a Mandarin-speaking female language teacher. For instruction purposes, the natural audio speech is recorded from the same native female language teacher, rather than using synthesized speech. The articulatory trajectories of 13 feature points included: Five facial feature points for calibration (nose, left head, right head, right jaw, and left jaw), four external feature points (right lip corner, and left lip corner, upper lip, lower lip) and another four internal feature points (tongue tip, middle tongue, tongue root, and middle jaw), which were normalized and smoothed. Then the static 3-D head model with tongue, uvula, and jaw was constructed based on anatomy that has great irregularity and a large number of vertices. Thus, a flexible graphic algorithm named Dirichlet free form deformation was adopted to compute the control parameters with Sibson coordinates for articulatory modeling work [29]. The EMA-based displacements were then added to the articulatory model, in order to drive multiple articulators to move smoothly and simultaneously over time. The articulatory model presented by this 3-D virtual tutor could depict realistic Mandarin articulation, giving an indication of the way of consonants and vowels being pronounced.

Second, many minimal pairs of Mandarin stops and affricates can be easily confused and are differentiated by the distinctive phonetic feature of unaspirated vs. aspirated contrast (e.g., b [p] vs. p [p‘]). These minimal pairs share the same place of articulation and similar trajectory of articulatory movement. To realize the visual information concerning the manner of articulation, exhaled airflow, one of the important bio-signals during speech production, was then collected and visualized to improve the identification rate of Mandarin consonants. Our recent study [41] collected airflow information with a *Phonatory Aerodynamic System* (PAS) and proposed an airflow model using a physical equation of the fluid flow. At the syllable-level animations, the new airflow model was then simultaneously combined with the 3-D articulatory model presented in [29], and a new multimodal animation system was constructed.

**Fig 1:**
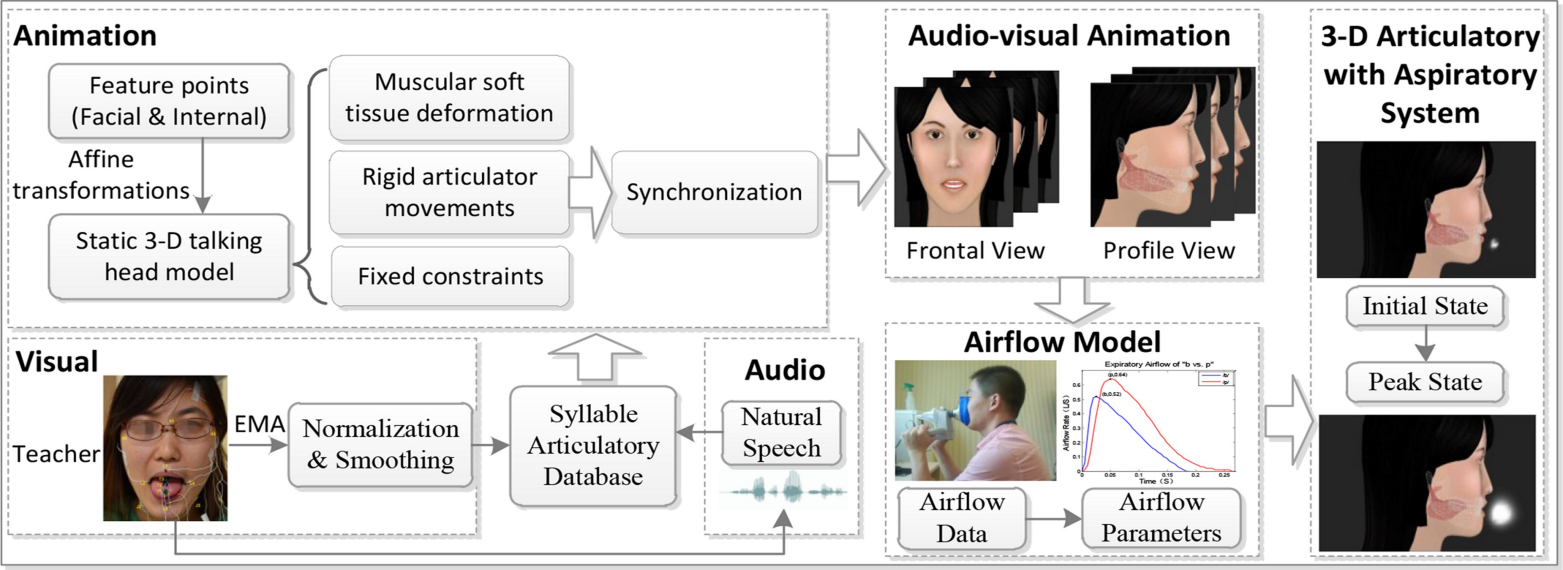
Implementation procedure for the 3-D virtual pronunciation tutor.

**Fig 2:**
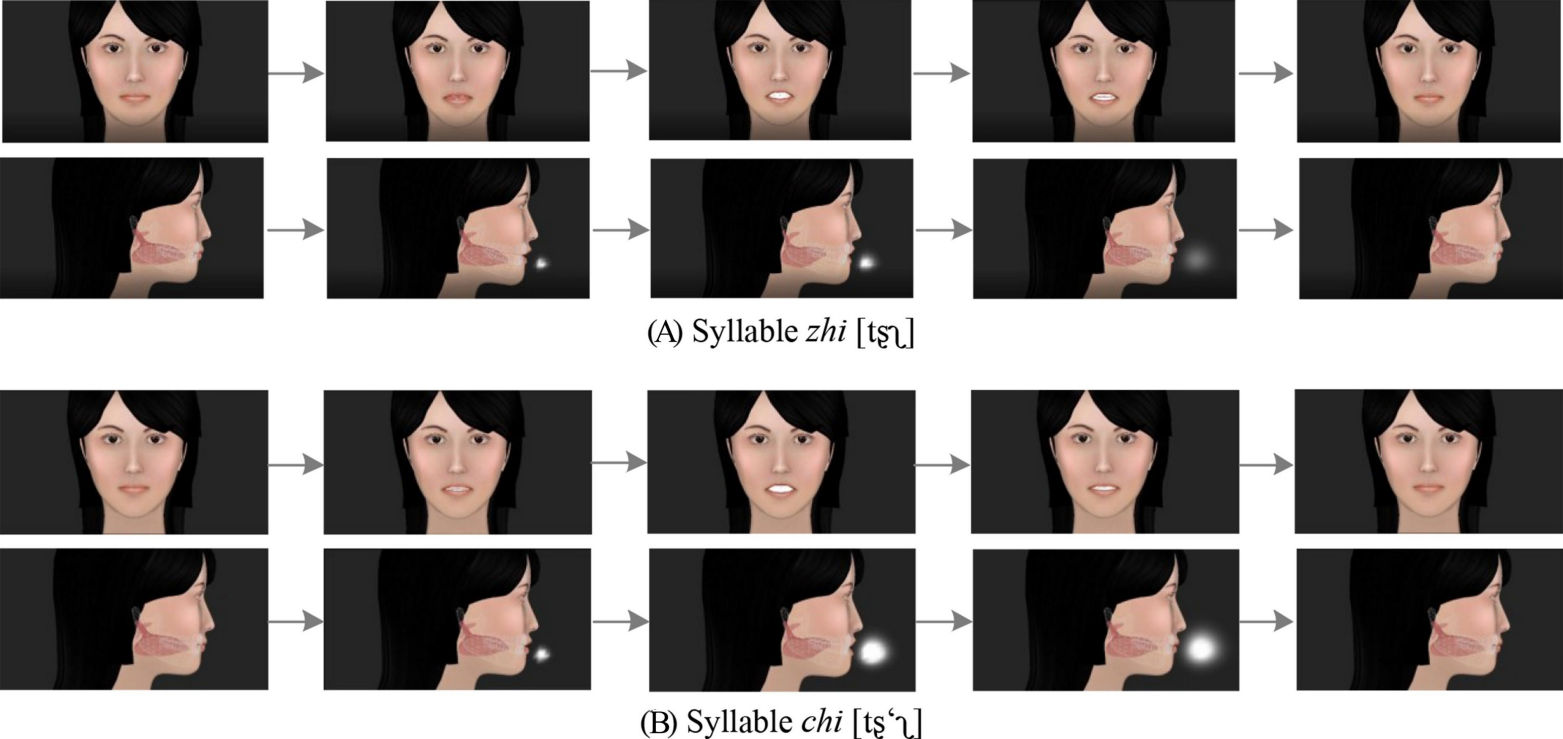
The 3-D articulatory with aspiratory animations for the sequence of two mandarin syllables. (A) zhi [tʂʅ], (B) chi [tʂ‘ʅ].

The overall implementation procedure of our 3-D pronunciation tutor is shown in Fig 1. This multimodal speech production tutor can present dynamic animations with both front and profile views. As shown in Fig 2, the 3-D virtual pronunciation tutor can show lip movements with a front view, and additionally, exhibit internal articulator (tongue) movements and airflow motions with a transparent profile view.

### Evaluation: Eye-tracking study (Experiment 1)

#### Participants

The participants included ten low-functioning children with ASD (eight boys) as an experimental group and 13 TD children (nine boys) as a control group. The average chronological ages of the two groups (see Table 1) were similar (*t* (21) = 1.07, *p* = 0.30, Cohen’s *d* = 0.42). All 23 child participants were native Mandarin speakers and had normal or corrected-to-normal visual acuity and normal hearing. Approval of the research was granted by Behavioral Research Ethics Committee of the Shenzhen Institutes of Advanced Technology, Chinese Academy of Sciences, and a written consent form was obtained from each child’s parent. The individual in this manuscript has given written informed consent (as outlined in PLOS consent form) to publish these case details.

**Table 1:** Chronological ages and developmental ages in child participants in Experiment 1.

Group	ASD (n = 10)		TD (Control) (n = 13)	
	Mean	SD	Mean	SD
Chronological Ages (Range, in years)	6.63	0.85	6.33	0.46
	(5.23–7.91)		(5.54–6.93)	
Developmental Ages (Range, in years)	3.15	0.71	N.A.	
	(2.24–4.83)		N.A.	

The clinical diagnosis of autism was established according to the DSM-V criteria for ASD [48], and further confirmed using the Chinese versions of the Gilliam Autism Rating Scale–Second Edition (GARS-2) [49] or the Childhood Autism Rating Scale (CARS) [50] by pediatricians and child psychiatrists with expertise in diagnosing ASD. The ASD participants in Experiment 1 showed different levels of language delay and speech production deficit based on speech-language pathology and parental reports, while none of them were non-verbal. Moreover, the *Psychoeducational Profile-Third Edition* (*PEP-3*) evaluation [51] showed that the average developmental age of expressive language and receptive language in ASD subjects was around 3.15 yr (SD = 0.71).

#### Apparatus

Eye movement data for each participant were collected with a *RED 5* Eye Tracker (SMI Technology, Germany) non-intrusively, which was integrated into a panel of 22-inch TFT monitor with a resolution of 1280×1024 pixels. The sampling rate was set to be 60 Hz and an accuracy of 0.4°. The freedom of head movement was 40 cm × 20 cm at 70 cm distance. Eye-tracking data were recorded online with the software of *Experiment Center 2*.*0* and analyzed offline with *SMI BeGaze* analysis software.

#### Testing materials

Training materials were comprised of 16 commonly used Mandarin syllables superimposed with high-level tone in Mandarin. They were combined with eight basic Mandarin monophthongs and 12 easily confused stops and affricates in terms of Pinyin (International Phonetic Alphabet (IPA) in the square brackets): bo ([po]), po ([p‘o]), bu ([pu]), pu ([p‘u]), de ([tɤ]), te ([t‘ɤ]), ga ([ka]), ka ([k‘a]), ju ([tɕy]), qu ([tɕ‘y]), ji ([tɕi]), qi ([tɕ‘i]), zi ([tsɿ]), ci ([ts‘ɿ]), zhi ([tʂʅ]), and chi ([tʂ‘ʅ]). All these 16 syllables were played under two presentation conditions (HF and 3-D tutors), totaling 32 videos, each containing a front view first and then a corresponding profile view. Videos in HF condition were time-aligned with those in 3-D condition with a duration of six seconds (three seconds for each view). The audio for each syllable was recorded from the same female speaker for both HF and 3-D tutors, and the volume was fixed at 75 dB SPL.

#### Procedure

**Fig 3:**
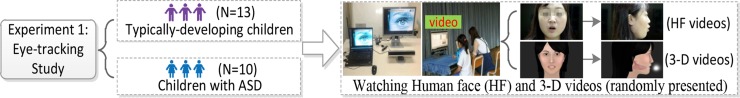
Procedure of the eye-tracking study in Experiment 1.

The procedure of Experiment 1 was shown in Fig 3. Firstly, a familiarization stage was undertaken to guarantee that all the child participants could follow the instructions. The experimenter taught them to place their chins above a fixed support frame, and to watch videos of two Mandarin syllables (ge [kɤ] and ke [k‘ɤ], excluded from the testing syllables) with two presentation conditions (i.e., HF and 3-D). While watching videos, subjects were asked to concentrate on what they heard and saw and imitate the pronunciation. However, the child participants in Experiment 1 were not told about the underlying meaning of visual contents to avoid any attention bias. Then, eye movement data were calibrated with five fixation points and recalibration was required if calibration results were poor or missing. Afterward, in the formal testing stage, the 32 testing videos were randomly repeated twice (totaling 64 videos) over four testing sessions.

#### Parameters of eye-tracking data

**Fig 4:**
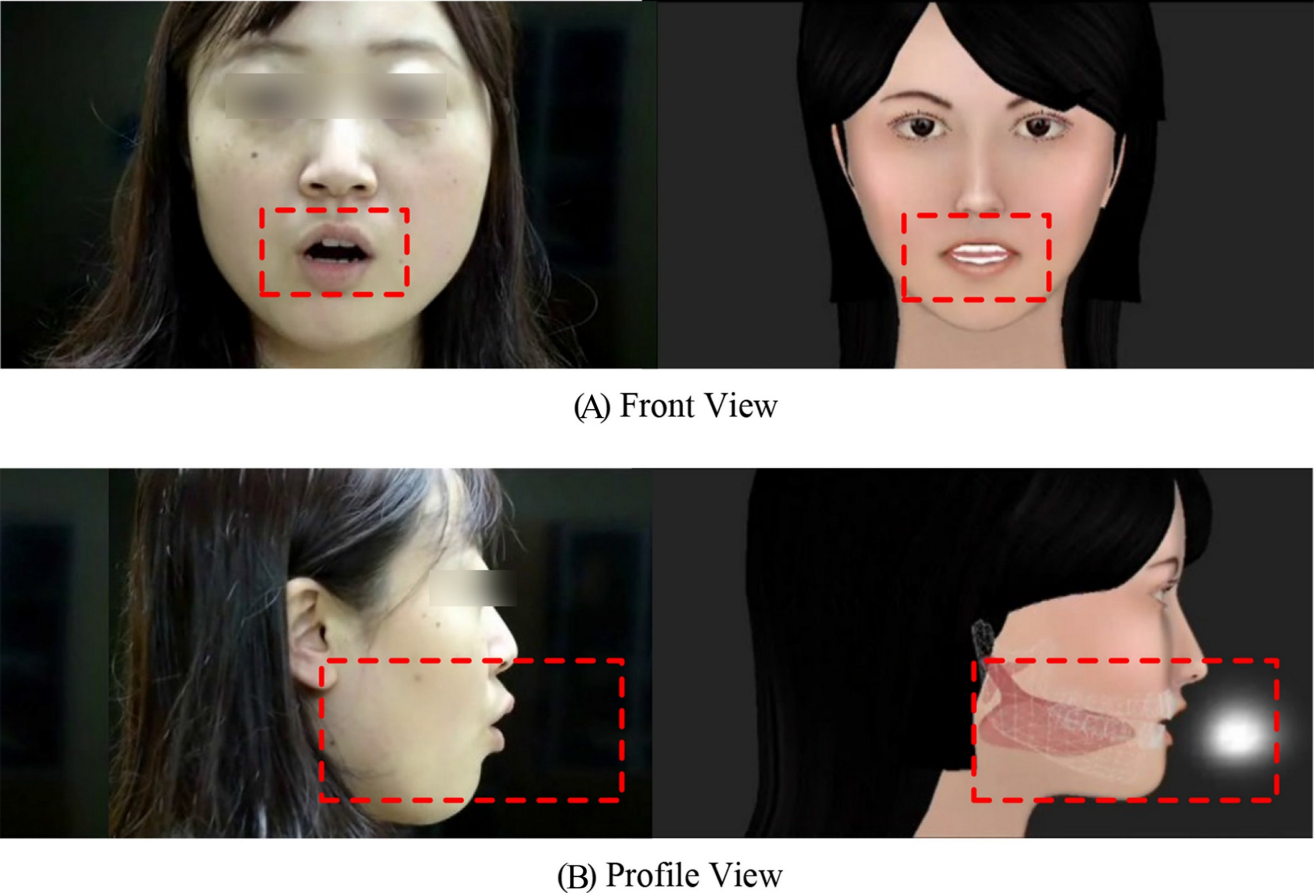
**The AOIs with a Front View (A) and a Profile View (B).** They corresponded to the homolographic areas inside the dashed red rectangle, which were closely related to speech sound production (with syllable po [p‘o] as an example). The AOI with a front view mainly incorporates lip movement area, and the AOI with a transparent profile view in the 3-D tutor contains the movement of external and internal articulators (including mouth, tongue, teeth, jaw, and palate) and airflow change.

With the front view (Fig 4A), the specified area containing lip movement was regarded as the area of interest (AOI). With the profile view (Fig 4B), the specified area in the 3-D video, including additional internal articulatory movements and airflow information, was regarded as the AOI. In the current study, three eye-tracking parameters were calculated during the learning process [52]. The first parameter was ‘entry time’ (ET), meaning the duration from the start of the trial to the first hit on the AOI. The shorter the ET, the more interest was shown for the AOI. The second parameter was ‘fixation count’ (FC), defined as the total number of fixations lasting more than 100 milliseconds (ms) inside the AOI, which reflects the absolute attention to the AOI during the learning process. The last parameter was ‘proportion of fixation duration’ (POFD), indicating the ratio of fixation duration inside the AOI to the duration of the whole video, reflecting relative attention to the AOI.

### Evaluation: Pronunciation training study (Experiment 2)

#### Participants

To avoid any practice effect from the first eye-tracking study when children were exposed to both HF tutor and 3-D tutor, a different 28 Mandarin-speaking children diagnosed with low-functioning ASD (chronological age range: 3.33–6.90 yr) were recruited to participate in the pronunciation training study. These ASD children were diagnosed with the same diagnostic approach as that used in Experiment 1. They were recruited in the current study since they all showed severe language delay and speech sound disorders based on both clinical observation and *The Psychoeducational Profile-Third Edition* (*PEP-3*) evaluation. Moreover, exclusion criteria included a history of hearing loss and exposure to more than one language in the child’s home. Not all the 28 children with ASD completed all tests and training. One child left the rehabilitation center during data collection, another three children were reluctant to cooperate with experimenters during the pre-test or post-test, and two children were dropped during the training period because they were not engaged by the computer screen and did not pay attention to the videos. These six child participants with ASD were thus excluded from any further analysis in the current study. Approval of the research was granted by Behavioral Research Ethics Committee of the Shenzhen Institutes of Advanced Technology, Chinese Academy of Sciences, and a written consent form was obtained from each child’s parent. The individual in this manuscript has given written informed consent (as outlined in PLOS consent form) to publish these case details.

In order to control for other possible factors that might influence training performance, all children with ASD completed the *PEP-3* evaluation, designed to assess the development of communication, motor skills, and presence of maladaptive behaviors in children with ASD [51]. The *PEP-3* was demonstrated to efficiently measure skills related to learning and to capture the uneven and idiosyncratic development of skills commonly found in children with ASD [53–55]. The *PEP-3* contains current normative data from a large representative sample of 407 individuals with ASD and a comparison group of 148 TD children. Moreover, the total raw score for all test items is converted into developmental ages (based on a TD sample). The related *PEP-3* evaluation results of developmental ages (including cognitive verbal/preverbal, expressive language, receptive language, fine motor, gross motor, and visual-motor imitation) in this study are presented in Table 2.

**Table 2:** The Means (standard deviations) of chronological ages, pre-test scores, and developmental ages of *PEP-3* tests in Experiment 2.

	CA (in years)	Pre-test Scores (1–5)		DA of *PEP*-3 tests (in year)					
		Consonants	Vowels	EL	RL	CVP	FM	GM	VMI
HF Group	4.87 (1.15)	3.42 (0.58)	3.63 (0.45)	2.23 (0.74)	2.30 (0.91)	2.46 (0.88)	2.33 (0.78)	2.24 (0.81)	2.30 (0.68)
3-D Group	4.81 (0.87)	3.39 (0.63)	3.61 (0.56)	2.33 (0.93)	2.30 (0.83)	2.30 (0.76)	2.51 (0.82)	2.38 (0.72)	2.27 (0.61)
*t*-value	0.13	0.13	0.09	-0.30	0.01	0.45	-0.54	-0.42	0.08
*p*-value	0.90	0.90	0.93	0.77	0.99	0.66	0.60	0.68	0.94

CA, chronological age; DA, developmental age; PEP-3, psychoeducational profile-third edition; EL, expressive language; RL, receptive language; CVP, cognitive verbal/preverbal; FM, fine motor; GM, gross motor; VMI, visual-motor imitation.

#### Testing materials and instrument

In both pre-intervention and post-intervention tests, all the child subjects were asked to imitate the audios containing the same 16 Mandarin syllables used in Experiment 1. The entire imitation task was recorded by Cool Edit software (22050 Hz sampling rate, 16-bit resolution) in a quiet aural rehabilitation room, and any vocalizations that occurred simultaneously with any other sounds on the recording were abandoned. Each syllable was imitated twice, with a total of 32 speech samples from each subject. For the audio imitation method, due to the presence of a speech model, the child’s true speech production abilities may have been overestimated. Nevertheless, in this special population, where speech output may be limited due to delayed language development, or a lack of desire to speak spontaneously, imitation may be the only option [18]. Moreover, one study [56] have indicated that the spontaneous and imitated production of speech in an ASD cohort did not differ in terms of sound class, and production errors tended to share both place and manner of articulation. Moreover, during pronunciation training, the computer-assisted 3-D or HF tutors were played through the software of an interactive speech training system [29].

#### Procedure and design

**Fig 5:**
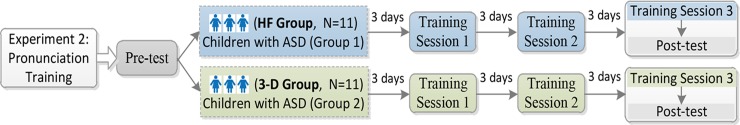
Procedure of the pronunciation training study in Experiment 2.

Fig 5 illustrates the procedure of Experiment 2. The performance comparison between pre-intervention test and post-intervention test was widely adopted to evaluate training outcomes in various pronunciation training studies [57,58]. Firstly, based on the average chronological ages, pre-test scores of consonants and vowels, and developmental ages of different *PEP-3* subtests, all the 22 children with ASD were equally divided into two subgroups (3-D group and HF group). Independent-samples *T* tests indicated that the two subgroups did not differ significantly in the above measurements (see Table 2). The 3-D group is the experimental group, while the HF group belongs to the control group. Each subgroup contained 11 subjects (one girl in each subgroup), and they were further asked to take part in a pronunciation training program which contains three sessions in total (see Fig 5). Within each session, the HF group learned the 16 syllables four times per session (three-day intervals between two consecutive sessions) from the HF tutors, while the 3-D group learned from the 3-D tutors with the same amounts of training. All the pronunciation tutors were presented as a front view first and then as a corresponding profile view, similar to that presented in Experiment 1. Each session lasted about an hour for each participant. During the training period (three repetitive sessions in total), learners were trained one by one in a quiet rehabilitation room, and they were asked to watch the videos in front of a computer screen and try to imitate pronunciation from the tutors afterwards. Besides, the ASD learners in Experiment 2 were taught the underlying meaning of various visual contents in tutors. To rule out a possible confounding effect of the daily routine training in the rehabilitation center, all the ASD learners’ instructors and speech therapists agreed not to additionally teach pronunciation during the training period. After the third training session, both subgroups were asked to conduct a post-intervention test with a similar audio imitation task to that in the pre-test (see Fig 5).

#### Scoring and inter-rater reliability

In this study, we collected and evaluated pre-test and post-test recordings to measure pronunciation performance. All the recorded speech samples were firstly rated by an expert majoring in linguistics to pick out the better trial from two iterations of each syllable. All the chosen 16 syllable production samples were then rated by another five Mandarin-speaking experts majoring in linguistics. Since all the tested Mandarin stops and affricates were voiceless, it is not appropriate to split the consonants and vowels and rate them separately. Raters were instructed to separately score each consonant and vowel production based on the perception of the heard phonemes and to minimize the mutual influence in their judgment of consonant or vowel production, even though they occurred next to each other within a carrying syllable. The 5-point Likert scale, with “1” (completely incorrect) and “5” (completely correct), was used to rate the quality of each phoneme production [58,59]. Different from previous studies with subjective judgements, the language experts in this study rated the speech tokens with a fine-grained criterion based on phonetic feature analyses. For example, a consonant b [p] was rated as “5” when the sound that was produced contained all the four phonetic features: voiceless, bilabial, unaspirated, and stop, while rated as “1” when none of the above four phonetic features were met. Moreover, to minimize transcribing bias, there was no identifiable information for each child (e.g., group, name, chronological age, gender) on the scoring sheets. To further minimize the experimental bias, raters did not know the experimental design and were blind as to which production data (pre-test or post-test) they were evaluating. Scoring for the 12 consonants and eight vowels from the 16 syllables was calculated and averaged for each ASD subject.

Inter-rater reliability was derived using SPSS (v.22.0). Kendall’s Concordance Coefficient W for the inter-rater agreement was calculated for scores derived from five raters. The inter-rater reliability with Kendall’s coefficients of 0.801 (consonant production in the pre-test), 0.896 (vowel production in the pre-test), 0.815 (consonant production in the post-test), 0.836 (vowel production in the post-test) were respectively reached for the scores given by the five experts, with all representing high inter-rater reliability.

## Results

### Experiment 1: Eye-tracking study

#### Entry time (ET)

**Fig 6:**
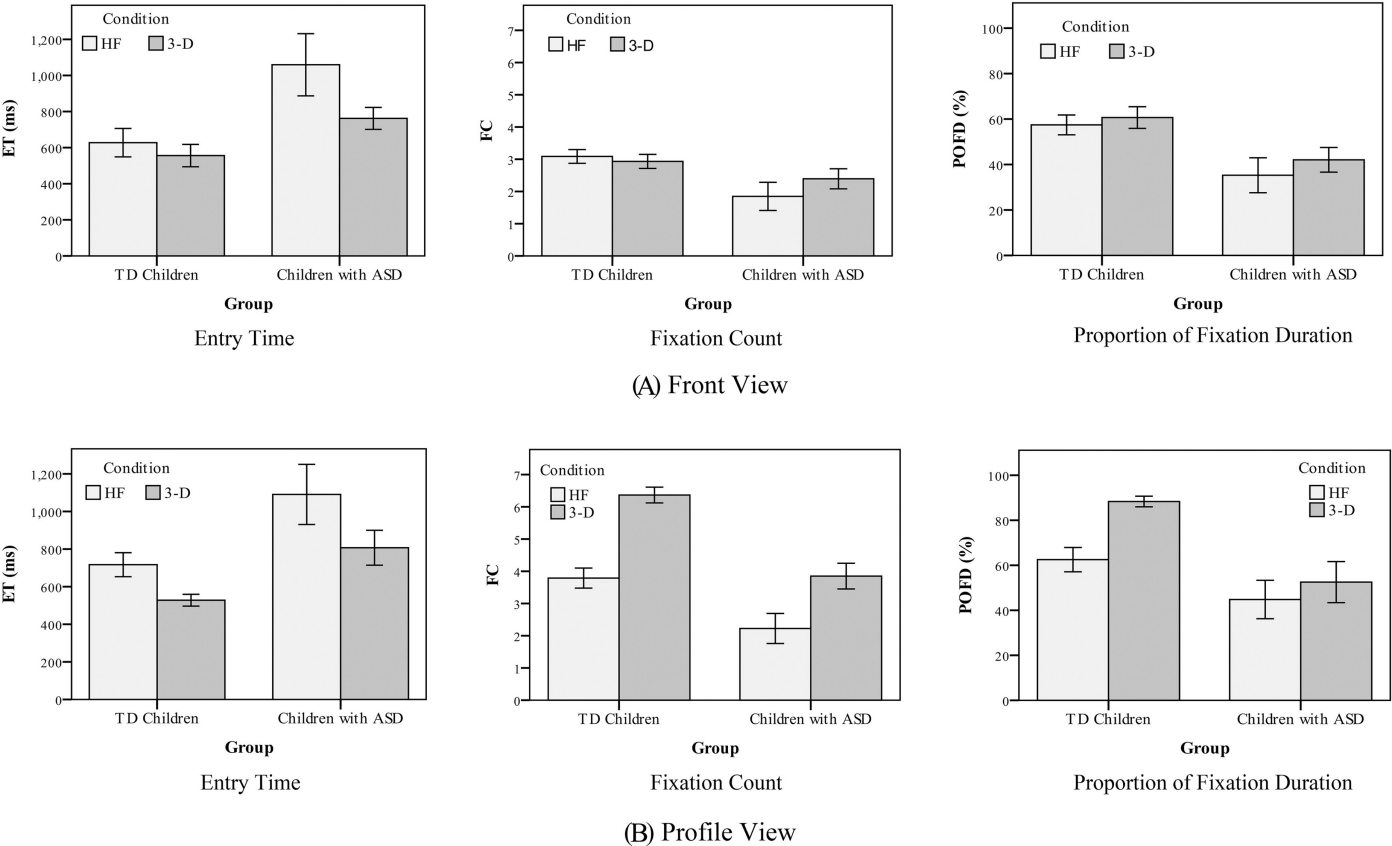
The results of three eye-tracking parameters. Average entry time (left), fixation count (middle), and proportion of fixation duration (right) of AOIs with a front view (A) and a profile view (B) in Experiment 1 (Error bars: +/- 1 SE).

All the eye-tracking data were analyzed in SPSS. Firstly, a three-way repeated-measure analysis of variance (ANOVA), with Greenhouse-Geisser corrections when appropriate, was conducted on the ET with *view* (front and profile) and *presentation condition* (HF and 3-D) as two within-subject factors, and *group* (ASD and TD) as a between-subject factor. The analysis revealed significant main effects for *presentation condition* (*F*(1, 21) = 7.28, *p* < 0.01, *η*_*p*_^2^ = 0.26), and *group* (*F*(1, 21) = 13.39, *p* < 0.001, *η*_*p*_^2^ = 0.39). The main effect of *view* was not statistically significant (*F*(1, 21) = 0.47, *p* = 0.50, *η*_*p*_^2^ = 0.02), and no significant two- or three-way interactions were observed (all *p*s > 0.05). The results indicated that all child subjects showed a shorter ET for the 3-D tutors with both front and profile views, and the TD children exhibited a much shorter ET than the children with ASD (see the left column in Fig 6).

#### Fixation count (FC)

Secondly, to examine the effect of *view* (front and profile), *presentation condition* (HF and 3-D), and *group* (ASD and TD) on the FC, a three-way ANOVA with repeated measures was conducted. Significant *view*×*presentation condition* interaction, *F*(1, 21) = 49.37, *p* < 0.001, *η*_*p*_^2^ = 0.70, and *view*×*group* interaction, *F*(1, 21) = 13.48, *p* < 0.01, *η*_*p*_^2^ = 0.39, as well as significant main effects of all three factors (all *p*s < 0.01), were observed. There was also a significant *view*×*presentation condition*×*group* interaction, *F*(1, 21) = 9.28, *p* < 0.01, *η*_*p*_^2^ = 0.31. This was followed up with a two-way analysis of factors *(presentation condition*, and *group*) with both front and profile views.

With a front view, the analysis confirmed a significant main effect for *group* on FC (*F*(1, 21) = 5.69, *p* < 0.05, *η*_*p*_^2^ = 0.21), while no main effect was detected for *presentation condition* on FC, such that similar absolute attention was paid to the AOI between HF and 3-D videos with a front view (*F*(1, 21) = 1.24, *p* = 0.28, *η*_*p*_^2^ = 0.06). There was no significant interaction between *presentation condition* and *group* (*F*(1, 21) = 3.94, *p* = 0.07, *η*_*p*_^2^ = 0.16) (see the middle column in Fig 6A). With a profile view, there were significant main effects for both *group* (*F*(1, 21) = 22.33, *p* < 0.001, *η*_*p*_^2^ = 0.52) and *presentation condition* on FC (*F*(1, 21) = 74.09, *p* < 0.001, *η*_*p*_^2^ = 0.78), while there was no significant interaction between *presentation condition* and *group* (*F*(1, 21) = 3.80, *p* = 0.07, *η*_*p*_^2^ = 0.15). The results indicated that both TD and ASD subjects paid more absolute attention to the AOI of 3-D videos with a profile view (see the middle column in Fig 6B). Furthermore, to better answer RQ2, the whole AOI in a transparent 3-D profile view was further divided into two subareas: Subarea 1 mainly contained an internal articulator showing places of articulation, and Subarea 2 mainly contained expiratory airflow showing manners of articulation. The parameter of FC was calculated to indicate the distribution of absolute attention paid to the two subareas in ASD learners. Results indicated that the average FC for Subarea 1 was approximately 2.20, and around 1.65 for Subarea 2 in children with ASD.

#### Proportion of fixation duration (POFD)

Thirdly, the eye-tracking parameter of POFD was submitted to a three-way ANOVA. The analysis revealed a significant *view*×*presentation condition*×*group* interaction, *F*(1, 21) = 5.14, *p* < 0.05, *η*_*p*_^2^ = 0.20. There were also significant main effects of *view*, *presentation condition*, and *group* (all *p*s < 0.01), as well as a significant *view*×*presentation condition* interaction (*F*(1, 21) = 6.06, *p* < 0.05, *η*_*p*_^2^ = 0.22). The significant three-way interaction indicated that the effects of *view* on the POFD were modulated by both *presentation condition* and *group*. This was followed up with a two-way analysis of two independent factors *(presentation condition* and *group*) with both front and profile views.

With a front view, the specified area containing lip movement was regarded as AOI, the main effect of *group* on POFD was significant (*F*(1, 21) = 7.78, *p* < 0.05, *η*_*p*_^2^ = 0.27), but the main effect of *presentation condition* on POFD was not significant (*F*(1, 21) = 3.27, *p* = 0.09, *η*_*p*_^2^ = 0.14). No significant interaction between *presentation condition* and *group* was found (*F*(1, 21) = 0.41, *p* = 0.53, *η*_*p*_^2^ = 0.02). This suggests that similar POFD (i.e., relative attention) was paid to the AOI of HF and 3-D videos with a front view (see the right column in Fig 6A). With a profile view, significant main effects of *presentation condition* (*F* (1, 21) = 16.82, *p* = 0.001, *η*_*p*_^2^ = 0.45) and *group* on the POFD (*F* (1, 21) = 10.69, *p* < 0.01, *η*_*p*_^2^ = 0.34) were found. Moreover, there was a significant two-way interaction between *presentation condition* and *group*, *F*(1, 21) = 4.90, *p* < 0.05, *η*_*p*_^2^ = 0.19. After this, simple main effect analyses of the *presentation condition* were performed with Bonferroni adjustment. For TD children, they showed a higher POFD towards 3-D videos with a profile view (*F*(1, 21) = 22.93, *p* < 0.001). However, for children with ASD, the *presentation condition* had no effect (*F*(1, 21) = 1.58, *p* = 0.22) (see the right column in Fig 6B).

**Fig 7:**
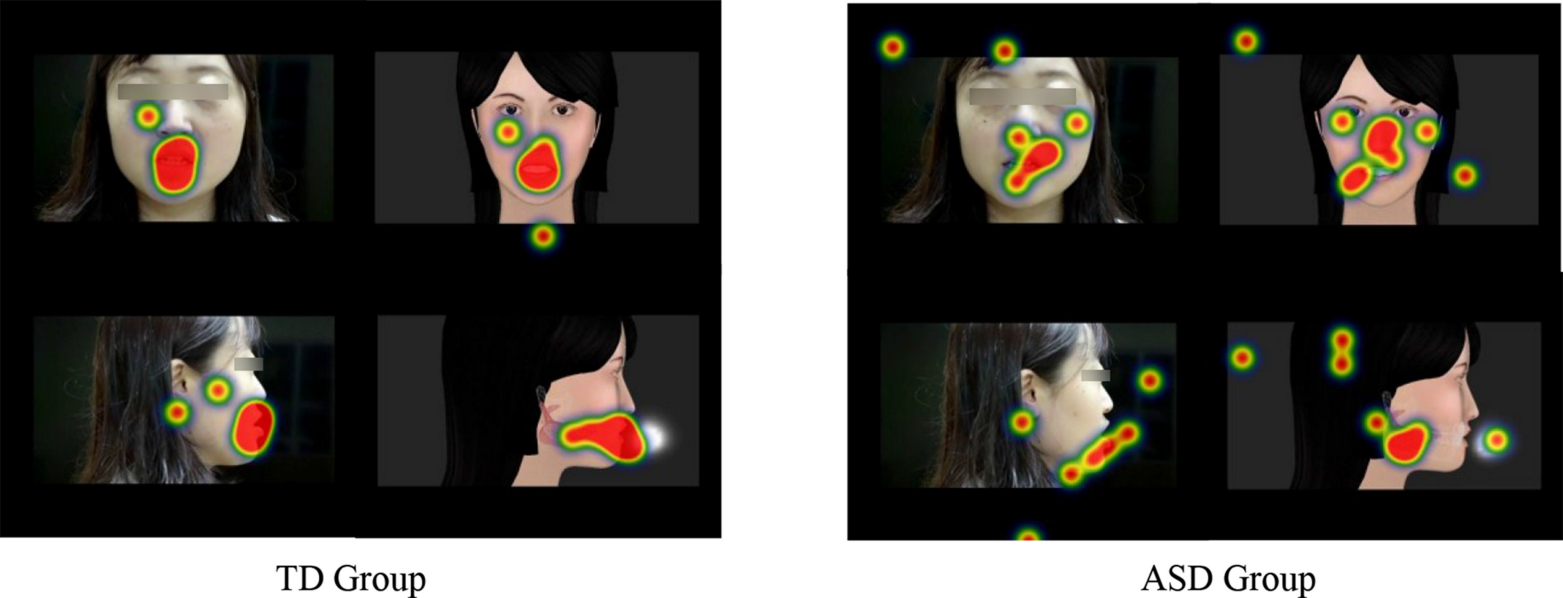
Heat maps of one testing syllable in Experiment 1.

Exploring the pattern of eye gaze in children is important to understand their attention distribution during learning. In Experiment 1, using the eye-tracking methodology, an objective evaluation comparing 3-D and HF tutors was conducted. RQ1 asked whether ASD learners showed interest in the AOIs in 3-D pronunciation tutor. Results showed that ET into the AOIs of 3-D videos was much shorter than that in the HF videos, indicating that children with ASD indeed showed more interest in the AOIs of our 3-D pronunciation tutor. The RQ2 asked whether ASD learners paid attention to additional articulation and airflow information with a 3-D profile view. The results showed that some fixation counts were indeed distributed to the motions of both internal articulators and aspirated airflow (also see the heat maps in Fig 7 for more detail). Finally, the RQ3 explored whether children with ASD showed a similar attention pattern to TD children. Although all the eye-tracking parameters indicated that, compared to TD children, ASD learners showed a much more scattered gaze behavior while watching the HF and 3-D videos, they still paid a relatively concentrated visual attention to the AOIs responsible for speech production (see Fig 7). In addition, like the TD children, ASD learners showed more interest in the AOIs in our 3-D virtual tutor.

### Experiment 2: Pronunciation training study

All the pronunciation data were analyzed in SPSS. The results of means and standard deviations of pre-test and post-test scores are listed in Table 3. Paired-sample *T* tests showed a significant improvement in the mean consonant scores for the HF group after training, *t*(10) = -4.46, *p* < 0.01, Cohen’s *d* = 0.65; a significant improvement in the mean consonant scores for the 3-D group, *t*(10) = -9.44, *p* < 0.001, Cohen’s *d* = 0.98; and a significant increase in mean vowel scores for the HF group, *t*(10) = -4.07, *p* < 0.01, Cohen’s *d* = 0.89; as well as a significant increase in mean vowel scores for the 3-D group, *t*(10) = -10.21, *p* < 0.001, Cohen’s *d* = 1.15.

**Table 3:** Pre-test and post-test scores in Experiment 2.

Group	Consonants					Vowels				
	Pre-test		Post-test		t-value	Pre-test		Post-test		*t*-value
	M	SD	M	SD		M	SD	M	SD	
HF Group	3.42	0.58	3.79	0.54	-4.46**	3.63	0.45	4.01	0.40	-4.07**
3-D Group	3.39	0.63	3.99	0.59	-9.44***	3.61	0.56	4.19	0.42	-10.21***

M, Mean; SD, Standard Deviation.

**p < 0.01

***p < 0.001

**Fig 8:**
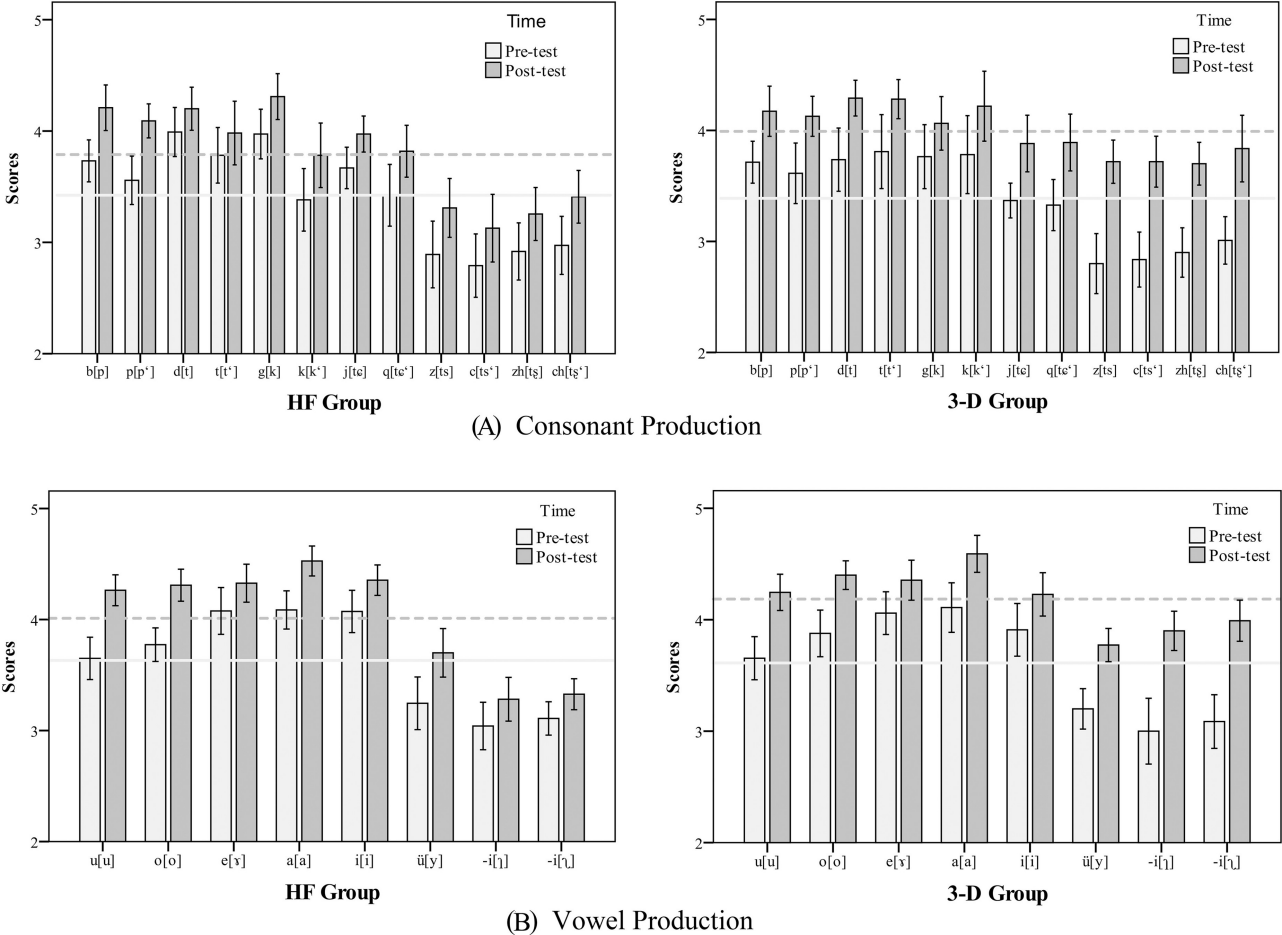
**The Pre-test and Post-test Scores for Different Mandarin Consonants (A) and Vowels (B) in HF Group (left) and 3-D Group (right) in Experiment 2.** The light solid lines indicate the average pre-test scores for different consonants or vowels, and the dark dashed lines refer to the average post-test scores (Error bars: +/- 1 SE).

The other key question of interest is whether the improvement in consonant or vowel scores from pre-test to post-test is greater for the 3-D group than it is for the HF group. The improvement (gain) from pre-test to post-test was computed for each participant by subtracting each person's pre-test score from his or her post-test score: *Gain score = post-test score–pre-test score*. First, a two-way 2 (*training group*: HF, 3-D) × 12 (*consonant*) ANOVA was conducted on gain scores for consonants, with the *consonant* as a within-subject factor and the *group* as a between-subject factor. The analysis confirmed that the 3-D group obtained higher gain scores for consonants than the HF group (*F*(1, 20) = 5.25, *p* < 0.05, *η*_*p*_^2^ = 0.21), while no main effect was found for *consonant* on gain scores (*F*(11, 220) = 0.86, *p* = 0.54, *η*_*p*_^2^ = 0.04). There was no significant interaction between *consonant* and *group* (*F*(11, 220) = 0.72, *p* = 0.65, *η*_*p*_^2^ = 0.03), (see Fig 8A and Table 4).

**Table 4:** Gain Scores of different mandarin consonants and vowels for HF and 3-D groups in Experiment 2.

Group	Consonants											
	*b*[p]	*p*[p‘]	*d*[t]	*t*[t‘]	*g*[k]	*k*[k‘]	*j*[tɕ]	*q*[tɕ‘]	*z*[ts]	*c*[ts‘]	*zh*[tʂ]	*ch*[tʂ‘]
HF	0.48	0.53	0.21	0.20	0.34	0.40	0.30	0.40	0.42	0.34	0.34	0.44
3-D	0.46	0.51	0.55	0.47	0.30	0.44	0.51	0.56	0.92	0.88	0.80	0.83

Group	Vowels							
	*u*[u]	*o*[o]	*e*[ɤ]	a[a]	*i*[i]	*ü*[y]	*-i*[ɿ]	*-i*[ʅ]
HF	0.61	0.54	0.25	0.44	0.28	0.45	0.24	0.22
3-D	0.59	0.52	0.30	0.48	0.32	0.57	0.90	0.90

Second, a two-way 2 (*training group*: HF, 3-D) × 8 (*vowel*) ANOVA was performed on gain scores for vowels, with the *vowel* as a within-subject factor and the *group* as a between-subject factor. Neither main effect of *vowel* (*F*(7, 140) = 1.74, *p* = 0.15, *η*_*p*_^2^ = 0.08) nor *group* (*F*(1, 20) = 3.17, *p* = 0.09, *η*_*p*_^2^ = 0.13) were significant. However, there was a significant interaction between *vowel* and *group*, *F*(7,140) = 2.50, *p* < 0.05, *η*_*p*_^2^ = 0.11. After this, a simple mean effect analysis was conducted with Bonferroni adjustment. For Mandarin vowels *u* [u], *o* [o], *e* [ɤ], *a* [a], *i* [i], ü[y], *group* had no effect (*all ps > 0*.*05*). However, for apical vowels *-i* [ɿ] (*F*(1, 20) = 9.48, *p* < 0.01) and *-i* [ʅ] (*F*(1, 20) = 16.69; *p* < 0.001), the 3-D group showed a relatively higher gain score than the HF group (see Fig 8B and Table 4).

In Experiment 2, a treatment outcome study was conducted to compare pronunciation training performance between two groups of children with ASD, learning from HF tutors or 3-D tutors respectively. Both groups showed significant improvements in consonant and vowel production from pre-test to post-test, after intensive computer-assisted pronunciation training. Moreover, ASD children learning from our 3-D pronunciation tutor tended to produce Mandarin stops and affricates and two Mandarin apical vowels better than those learning from the HF tutor. It is, therefore, reasonable to conclude that children with ASD can benefit more from our 3-D virtual pronunciation tutor.

## Discussion

The main goal of our investigation was to compare the treatment efficacy of a HF tutor and our 3-D virtual pronunciation tutor for pronunciation intervention in low-functioning children with ASD. These two types of tutors (HF tutor and 3-D tutor) were widely used in various computer-assisted pronunciation training systems [33,58,60]. The results of the first eye-tracking study implied that, during the learning process, ASD learners showed more interests in the AOIs of 3-D virtual tutor and paid some degree of absolute attention to the additional visual speech cues of articulatory and airflow models in our 3-D virtual tutor. The learners’ visual attention to visual speech cues was important for language learning as the ‘noticing hypothesis’ [61] indicates that noticing itself does not result in acquisition, but is an essential first step in acquiring a speech item. In experiment 2, a treatment outcome study was further conducted and showed that, compared with those learning from HF tutors, the 3-D group showed a much higher increase in scores while uttering Mandarin stops, affricates, and two Mandarin apical vowels (-i[ɿ], -i[ʅ]). Based on these findings, we could conclude that relative to the HF tutor, children with ASD benefited more from noticing the visual speech cues in our multimodal 3-D pronunciation tutor. Furthermore, the user experience was evaluated by conducting a short oral interview with parents or caregivers after pronunciation training, which included three aspects: enjoyment, motivation, and acceptability. Although the enjoyment level was medium with some degree of concerns about the uncommon internal articulators in daily life, most of the parents or caregivers showed a strong motivation and a high acceptability towards our computer-assisted 3-D virtual pronunciation tutor, and even wanted to make a copy of the 3-D videos. To conclude, the above quantitative and qualitative results showed the benefit and usability of the implementation of 3-D virtual tutor for the pronunciation training in children with ASD.

The underlying learning mechanism of our current approach was built on ‘imitation learning’ [24,25], which relies on a straightforward and realistic presentation of key acoustic features from our 3-D virtual tutor. The mirror system based therapy has been proved to be effective in several robot-mediated training systems for learning by imitation [62–64], showing that robot-mediated imitation learning for children with ASD was effective and produced relatively better performances than a human therapist. In the current study, our multimodal 3-D pronunciation tutor with a transparent profile view can realistically exhibit synthetic visual speech with bio-data-driven external and internal articulatory movements (i.e., the place of articulation), and expiratory airflow information to discriminate Mandarin aspirated from unaspirated stops and affricates (i.e., the manner of articulation). Furthermore, the visual cues to the contrast between two Mandarin apical vowels were sufficiently salient and easily discriminated by observing the apical motion from front (-i[ɿ]) to back (-i[ʅ]) in our 3-D tutor. Mandarin-speaking children with ASD in our investigation benefited from noticing these visual speech cues through repeated exposure, and their intended learning by imitation from additional visual speech cues in our 3-D virtual tutor could partly explain the better training outcomes in children with ASD. Learning by imitation requires little priori linguistic knowledge, making this methodology suitable even for young children and individuals with severe cognitive disorders. These findings implicate a new approach in pronunciation training for ASD learners, by pedagogically illustrating visual speech cues in a 3-D virtual tutor.

The possible theoretical significance of this study is shown as follows. Firstly, our findings may provide complementary evidence that children with ASD, although somewhat restricted in their ability to use visual information from a tutor, can integrate visual and vocal information and further improve their skill of speech production. These findings are consistent with previous studies [65,66] which indicated that while children with ASD were less accurate in recognizing stimuli in a unimodal condition, they showed a normal integration of visual and auditory speech stimuli. The additional visual information related to speech sound production in our 3-D tutor is likely to be of great importance to audiovisual pronunciation training for children with ASD. Secondly, another interesting theoretical speculation [67,68] indicated that, in gaze behavior patterns looking at the eyes and mouth during face perception, children with ASD look more towards the mouth region because they tend to orient toward audiovisual synchrony. Children with ASD may focus on the mouth initially because of its physically contingent properties, seeing the world in terms of its physical features rather than its social-affective context. This theoretical speculation suggests an alternative learning path for language acquisition: language skills are being acquired with the help of physical features (the relationship between motion and sound) rather than social-affective features (speech sounds as social cues). Similarly, in our 3-D virtual tutor, when the external and internal articulators and airflow began to change, the speech sounds started to play; when the speech sounds stopped, the animations also ceased. Children with ASD in our study tended to concentrate attention on the motion of articulators and airflow changes, probably due to their synchrony with speech sound, and this process may account partly for the greater improvement in pronunciation scores with the help of a 3-D virtual tutor.

With respect to the practical significance, our computer-assisted 3-D pronunciation tutor can be utilized remotely at home or in the community, potentially decreasing the number of in-person intervention hours that ASD children would need to have with speech-language pathologists (SLPs). Since there is a short of SLPs and therapists especially in developing countries [69], our cost-effective 3-D virtual tutor could be used as one of the substitutes which could potentially reduce the ASD family's financial burden. Generating synthetic visual speech through the 3-D virtual tutor can provide a novel mode of pronunciation training and repeatedly provide ASD learners with one-to-one instruction anytime and beyond the traditional classroom environment in a rehabilitation center, which can make a significant difference from traditional language learning methods [2,19–23].

Furthermore, an increasing number of recent studies have suggested the great benefit of computerized technologies as therapeutic and educational tools for individuals with ASD [70–72]. In the current study, we also found benefits in utilizing the methodology of computer-based pronunciation training for children with ASD. In the eye-tracking study, while watching the HF or 3-D videos presented on a computer screen, ASD learners paid relatively concentrated visual attention to the AOIs which are closely related to speech production. In Experiment 2, with a short-term and intensive computer-based pronunciation training package, we found convincing evidence that production enhancement was indeed occurring with the computer-based program. Both 3-D and HF groups showed gains in consonant and vowel production from pre-test to post-test. These results are encouraging, and are in line with other positive treatment outcomes with computer-based instruction, showing it to be an effective method to train and develop vocabulary and reading knowledge in children with ASD [34,73–75]. As mentioned previously in this paper, failure to attend to the ambient language environment hinders the ability to acquire spoken language in children with ASD, and also leads to a reduced tendency to hone speech sound production from speech models produced by others in the social environment. Presenting learning materials of HF or 3-D tutors via the computers can potentially diminish the social difficulties for some children with ASD when interacting with a teacher or an SLP.

Computer-based 3-D virtual pronunciation training for ASD learners might be of great help, but some caution should be exercised before overstating this claim. As mentioned in Experiment 2, a small proportion of learners with ASD were dropped during the training period. Regardless of our coaxing and persuading, they were not attracted to the computer. This phenomenon is somewhat understandable given that the behavioral difficulties widely observed in children with ASD, such as lack of cooperation, and resistance to novel methods, often create difficult situations that are not optimal for learning [76]. However, for these small groups of ASD children, consequently, other styles of learning should be explored and supported. We need to be more ingenious in capturing ASD learners’ attention. For examples, in future studies, the technique of automatic speech recognition could be integrated into the current 3-D virtual tutor, to evaluate the ASD learners’ pronunciation online and give feedback in time to better enhance interaction and attract the ASD learners’ attention. Moreover, as suggested by [77], a child does not progress by acquiring units like phonemes or allophones, but rather by gradually adding lexical items to his/her repertoire. Consequently, the process of phonology acquisition in the early stages is not phoneme by phoneme, but word by word. In the future, we might make use of the treatment of specific speech sounds as a catalyst or stimulus to the word level, and combine phonology and lexicon learning together. In this way, certain speech sounds would be related to a certain word. The concurrent presentation of a 3-D virtual pronunciation tutor and a corresponding word picture with varying colors and shapes may be more engaging and motivating for children with ASD. In short, an ideal pronunciation training system should be individually tailored for each ASD student to put them in a good mood and encourage them to become more interested in working with the computer to enjoy imitating the speech sounds they hear and see.

## Conclusions

A subgroup of children with ASD, especially those with more severe global language impairment, may exhibit more severe speech sound production difficulties. Clinicians and SLPs should be aware that children with more severely impaired language and behavior, may exhibit more severe speech production difficulties. Recently, two studies [37,78] have emphasized the critical need for both researchers and clinicians to address pronunciation problems and to focus on speech sound behavior in individuals with ASD. However, available interventions that aim to improve pronunciation ability in children with ASD are extremely limited.

In this study, a computer-based 3-D virtual pronunciation tutor was proposed and evaluated. The current findings indicated that individuals with ASD who are struggling with speech sound production could benefit more from our 3-D pronunciation tutor exhibited on a computer screen. By demonstrating additional visual information during speech sound production, our 3-D virtual speech production tutor provides an efficient pronunciation training method to enhance consonant and vowel production skills among the ASD cohort. We advocate multimodal learning environments to enhance speech production and other language skills among an ASD cohort.
